# Deep learning-assisted diagnosis of liver tumors using non-contrast magnetic resonance imaging: a multicenter study

**DOI:** 10.3389/fonc.2025.1582322

**Published:** 2025-07-10

**Authors:** Shihui Zhen, Peng Zhang, Hanxiao Huang, Zhiyu Jiang, Yankai Jiang, Jihong Sun, Liqing Zhang, Mei Ruan, Qingqing Chen, Yujun Wang, Yubo Tao, Weizhi Luo, Ming Cheng, Zhetuo Qi, Wei Lu, Hai Lin, Xiujun Cai

**Affiliations:** ^1^ Department of Surgical Oncology, Sir Run Run Shaw Hospital, School of Medicine, Zhejiang University, Hangzhou, Zhejiang, China; ^2^ Department of General Surgery, Sir Run Run Shaw Hospital, School of Medicine, Zhejiang University, Hangzhou, Zhejiang, China; ^3^ State Key Laboratory of Computer-aided Design and Computer Graphics (CAD&CG), Zhejiang University, Hangzhou, Zhejiang, China; ^4^ Shanghai Artificial-Intelligence Laboratory, Shanghai, China; ^5^ Department of Radiology, Sir Run Run Shaw Hospital, School of Medicine, Zhejiang University, Hangzhou, Zhejiang, China; ^6^ Department of Radiology, Affiliated Hangzhou First People’s Hospital, Zhejiang University School of Medicine, Hangzhou, China; ^7^ Department of Radiology, Tongde Hospital of Zhejiang Province, Hangzhou, China; ^8^ Department of Hepatobiliary and Pancreatic Surgery, First Affiliated Hospital, Zhejiang University School of Medicine, Hangzhou, China; ^9^ Department of Radiology, Ningbo NO. 2 Hospital, Ningbo, Zhejiang, China

**Keywords:** deep learning, liver tumor, classification, non-contrast, magnetic resonance imaging

## Abstract

**Objectives:**

Non-contrast MRI(NC-MRI) is an attractive option for liver tumors screening and follow-up. This study aims to develop and validate a deep convolutional neural network for the classification of liver lesions using non-contrast MRI.

**Methods:**

A total of 50418 enhanced MRI images from 1959 liver tumor patients across three centers were included. Inception-ResNet V2 was used to generate four models through transfer-learning for three-way lesion classification, which processed T2-weighted, diffusion-weighted (DWI) and multiphasic T1-weighted images. The models were then validated using one independent internal and two external datasets with 5172, 2916, and 1338 images, respectively. The efficacy of non-contrast models (T2,T2+DWI) in differentiating between benign and malignant liver lesions at the patient level was also evaluated and compared with radiologists. The performance of models was evaluated using the area under the receiver operating characteristic curve (AUC),sensitivity and specificity.

**Results:**

Similar to multi-sequence and enhanced image-based models, the non-contrast models showed comparable accuracy in classifying liver lesions as benign, primary malignant or metastatic. In the independent internal cohort, the T2+DWI model achieved AUC of 0.91(95% CI,0.888–0.932), 0.873(0.848-0.899), and 0.876(0.840-0.911) for three tumour categories, respectively. The sensitivities for distinguishing malignant tumors in three validation sets were 98.1%, 89.7%, and 87.5%%, with specificities over 70% in all three sets.

**Conclusions:**

Our deep-learning-based model yielded good applicability in classifying liver lesions in non-contrast MRI. It provides a potential alternative for screening liver tumors with the advantage of reducing costs, scanning time and contrast-agents risks. It is more suitable for benign tumours follow-up, surveillance of HCC and liver metastasis that need periodic repetitive examinations.

## 1 Introduction

Liver cancer is one of the leading causes of cancer-related mortality worldwide (1). Based on the primary tumor site, liver cancer may be divided into primary liver cancer and metastatic cancer of the liver. Hepatocellular carcinoma (HCC) accounts for 75-85% of primary liver cancer while intrahepatic cholangiocarcinoma (ICC) accounts for 10-15% (2). The liver is also the dominating site of metastasis for gastrointestinal cancers and is a location highly susceptible to the establishment of metastasis in many other primary cancers, including breast, lung, and pancreatic cancers (3). In addition, several types of benign masses also arise in the liver, including cyst, hemangioma, focal nodular hyperplasia, abscess and some benign nodules, such as cirrhotic nodules, regenerative nodules, dysplastic nodules and adenoma (4, 5). Clinically, a key diagnostic challenge lies in differentiating between primary hepatic malignancies, metastatic lesions, and benign tumors. While benign, asymptomatic lesions typically require no intervention other than observation (6),accurate and timely diagnosis of malignant liver lesions is crucial for effective treatment and improved prognosis (7).

Compared to ultrasound and computed tomography (CT), Magnetic Resonance Imaging (MRI) achieves higher detection rate and diagnosis accuracy for focal liver lesions, which makes it the best candidate for surveillance of liver cancer (8, 9). However, full contrast-enhanced MRI protocols are limited by long acquisition times, high costs, and the potential adverse effects of gadolinium-based contrast agents, including nephrogenic systemic fibrosis and gadolinium deposition in tissues (10–19).

Non-contrast MRI (NC-MRI), incorporating T2-weighted (T2W) imaging and diffusion-weighted imaging (DWI), is emerging as a practical and safer alternative, especially for patients requiring repeated follow-up. HCC presents with mild to moderate hyperintensity on T2-weighted images, while non-malignant lesions (e.g. cysts, hemangiomas, fibrosis) usually display marked T2 hypo-intensity or marked T2 hyperintensity (20). However, NC-MRI still has some limitations. Lesions like FNH and adenomas can mimic malignancy, and certain HCCs may appear isointense to the surrounding liver parenchyma on T2WI. DWI is vulnerable to artifacts and has blind spots. Some reviews pointed out that relatively low sensitivity and low inter-reader agreement are main concerns in NC-MRI (21, 22).

With the advancement of artificial intelligence in medical imaging, deep learning (DL), particularly convolutional neural networks (CNNs), has shown great promise in improving image-based diagnosis (23, 24). Although several studies have applied DL to liver lesion classification, most of them rely on contrast-enhanced MRI, limiting their applicability in routine screening or contrast-contraindicated patients (25–28). If CNN-based DL models can achieve high diagnostic performance using only NC-MRI, this would substantially reduce the cost and complexity of liver tumor surveillance, while minimizing patient risk. This would be especially advantageous for patients with benign lesions requiring long-term follow-up and for those under regular surveillance for HCC or liver metastases. Therefore, this study aims to evaluate the diagnostic performance of a deep learning model using only non-contrast MRI for classifying liver tumors. Specifically, we developed and validated the model on a multicenter dataset encompassing diverse liver lesion types, and compared its performance with that of experienced radiologists to assess its clinical utility.

## 2 Materials and methods

### 2.1 Study design

**Figure 1:**
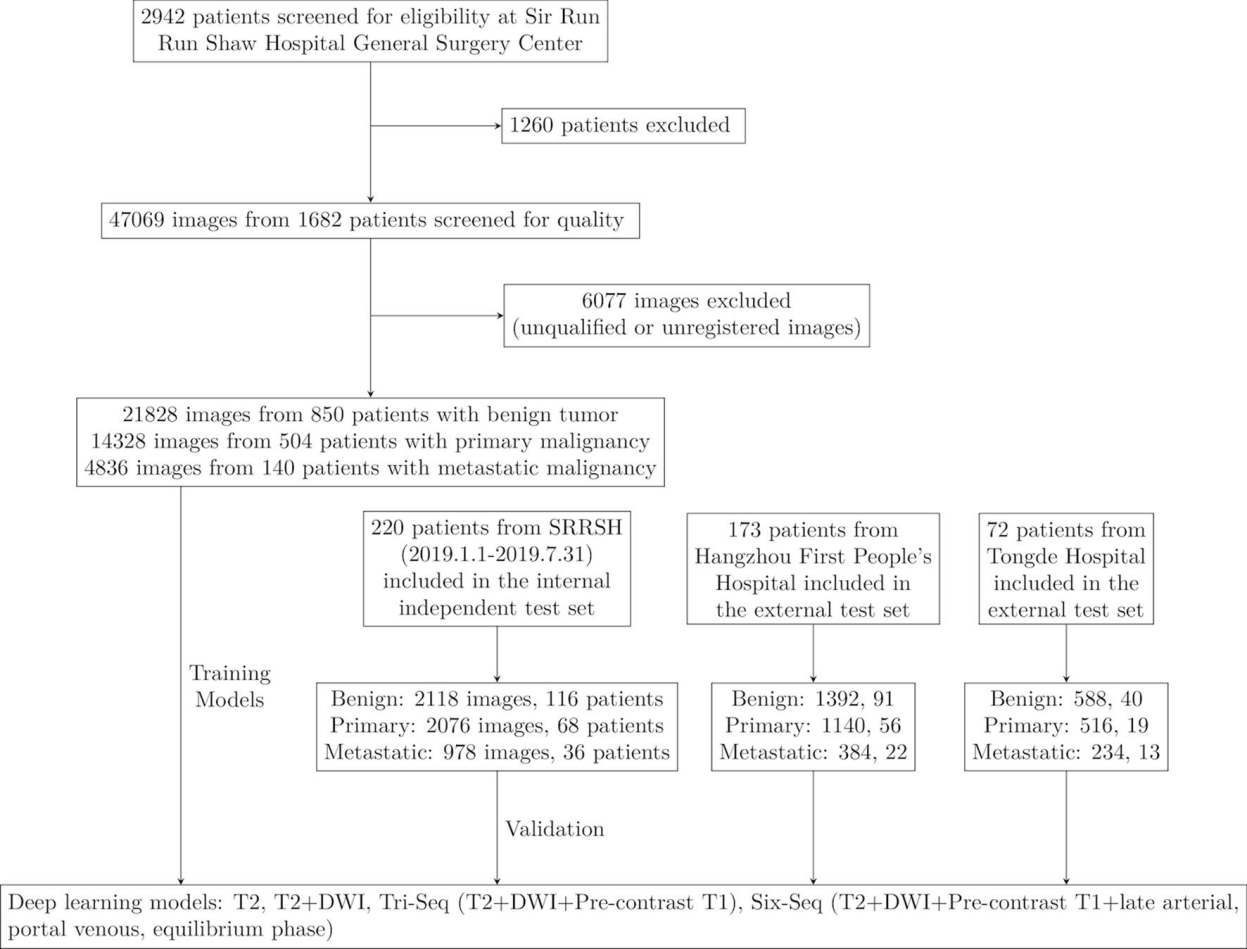
Workflow diagram for the development and evaluation of deep learning models. SRRSH, Sir Run Run Shaw Hospital.

This was a retrospective, multi-center, diagnostic study using liver MRI image sets from three hospitals in China. The inclusion criteria were as follows: (1) with liver tumors; (2) accepted enhanced MRI inspection; (3) with final diagnosis: histopathologic report from biopsy or surgery; HCC with typical Li-RADS 5 imaging diagnostic criteria; metastatic lesions with typical imaging features and known primary sites; benign tumors with typical imaging features; (4) aged 18 years or older. The exclusion criteria were as follows: (1) accepted treatment related to the lesion before MRI inspection, including surgery, transcatheter arterial chemoembolization (TACE), radiofrequency ablation, chemotherapy, radiotherapy, targeted drug therapy, etc.(2) unqualified image quality. This study consisted of two stages: the training stage, in which deep learning models were trained using MRI image sets from the hepatic focal lesion database by affiliated Sir Run Run Shaw Hospital, Zhejiang University School of Medicine (SRRSH, obtained from January 2014 to December 2018) and the test stage to examine the performance of the models using three different test MRI image sets which were obtained from SRRSH (January 2019 to July 2019), Hangzhou First People’s Hospital (HZFPH) and Tongde Hospital of Zhejiang Province(TDH), respectively. In the training, to classify liver tumors into three categories, we undertook a series of supervised CNN learning using different combinations of MRI sequences (T2, diffusion, Pre-contrast T1, late arterial, portal venous, equilibrium phase) as input data. A flowchart of the outline of this study are demonstrated in **Figure 1**.

This study has been approved by the Institutional Review Board of Sir Run Run Shaw Hospital (SRRSH) and was conducted in accordance with the Declaration of Helsinki. This work has been reported in line with the STARD (Standards for the Reporting of Diagnostic accuracy studies) criteria (29).

### 2.2 Ground truth

Four general radiologists with more than 10 years of experience in abdominal imaging diagnosis were divided into 2 groups of 2 to participate in data quality control and data annotation. Each lesion was manually annotated by two general radiologists, with one radiologist delineating the boundaries of the lesion under the supervision of another radiologist. The contours of the lesion were finalized when the two radiologists reached a consensus.

The gold standard for lesion classification was established either from available histopathological reports or from the consensus of two senior general radiologists, each with over 20 years of experience in abdominal imaging diagnosis. Specifically, malignancies were validated via histopathology, while benign lesions were confirmed either through appropriate histopathology or by the joint agreement of the senior radiologists mentioned earlier. The agreement was achieved after an independent review of all pertinent information, which included clinical data, MRI scans, and associated radiological reports which were collected over a follow-up period of at least six months. Cases that had neither a histopathological report nor a consensus agreement were all excluded from the study. For patients who had several liver masses of the same diagnosis, the most typical and largest liver mass was selected. These datasets covered almost all types of liver mass-like lesions.

Liver masses were finally classified into three categories adhering to the criteria as follows: A. benign tumor, including these types: cyst, hemangioma, abscess, focal nodular hyperplasia (FNH), other benign nodules (cirrhotic nodules, regenerative nodules(RN), dysplastic nodules(DN), rare benign tumors); B. primary malignancy, including HCC and other primary hepatic malignancy(intrahepatic cholangiocarcinoma(ICC), mixed HCC-ICC, etc.); C. metastatic malignancy, with primary sites from colorectal, breast, lung, pancreas, etc.

### 2.3 MRI acquisition protocol

Abdominal MRIs were performed in the supine position. The T2 weighted sequence and diffusion weighted sequence (b value: 800s/mm^2^ or 1000s/mm^2^) were performed according to the standard institutional liver MR imaging protocol, and the acquisition time was 2-2.5min and 2-2.5min, respectively. Contrast-enhanced T1 sequences were performed with acquisition time of 12–18 s. Images of pre-contrast T1, late arterial phase (~ 15s post-injection), portal venous phase (~ 60 s post-injection) and equilibrium phase (~3 min post- injection) were also screened. The scanners and contrast media used for MR acquisition in three hospitals are listed in **Supplementary Table S1**. Imaging parameters varied across different scanners and time frames.

### 2.4 Image preprocessing

Eligible MRI images were downloaded from the Picture Archiving and Communication Systems (PACS) and stored as Digital Imaging and Communications in Medicine (DICOM) files. The region of interest (ROI) about liver tumor was annotated in T2 sequences by trained senior abdominal radiologists based on ground truth standard. Six images from six sequences (T2, diffusion, Pre-contrast T1, late arterial, portal venous, equilibrium phase) were then obtained for each cross section of the lesion and resampled to a resolution of 0.7 × 0.7 × 10 mm. Then the annotations of the other five sequences were generated according to the origin and spacing information of sequences. DICOM files were converted to images for the training stage. To increase the diversity of data, the images were augmented using rotation, flipping, scaling, shifting and shearing.

### 2.5 Deep learning model development

The overall process of the proposed deep learning system to liver tumor diagnosis is explained in **Figure 1**. Our network architecture was initially derived from Google Inception-ResNet V2 CNN architecture. For initializing the network, we applied a transfer learning method with backbone network pretrained on ImageNet dataset (30) (see **Supplementary Figure S1**), while the first convolution layer was modified to take in inputs of three or six channels (for a single T2 sequence input, the T2 images were copied and stacked to have three identical channels; for multi-sequence input, the sequences were stacked in specified orders), and the last fully-connected layer was modified to output three channels (for tri-classification task) or two channels (for binary-classification task). For each group of input images, the output was a three or two-dimensional vector representing the predicted probabilities for the three or binary categories. The category with the largest value in the vector was taken as the predicted diagnosis. To calculate the patient-wise predicted value, the predicted vector for each image group was summed up and the category with the largest value was used as the final diagnosis of the patient.

The network was trained via back-propagation. The optimization was stochastic gradient descent with global learning rate of 0.1 and momentum of 0.9, while the step decay was set to decrease by 50% every 20 epochs, combined with a linear warm-up in the first 10 epochs. The training epoch was set to be 200 and batch size as 16. Python and TensorFlow framework were used to implement the training and validation stages. During the training and validation stages, each image was first resized to 299×299 pixels with bicubic interpolation. The images were also augmented via random rotation within 40°, horizontal/vertical flip, and width/height scaling, shearing and zooming which were all within 20%.

All codes were implemented in Python and Pytorch. One work-station was used for individual model training and validation. More specifically, all experiments were performed on a workstation platform with 2 NVIDIA RTX 2080 Ti GPUs with 11GB GPU memory, 256 G RAM, 1 NVIDIA RTX 1080Ti GPU and Intel(R) Xeon(R) Gold6248 CPU @ 2.50 GHz, using Ubuntu 16.04.

To generate a visual explanation of the model diagnosis process, attention maps were plotted using the Grad-CAM algorithm which displayed the pixels in the ROIs that provided the greatest contribution to the classification output (31).

### 2.6 Statistical analysis

Descriptive statistics were summarized as mean ± SD. Comparisons between groups were made with the Kruskal-Wallis H test, when appropriate, for quantitative variables and with the X2 test or Fisher’s test for qualitative variables. For classification purposes, the receiver operating characteristic (ROC) curve was used to show the diagnostic ability of the model in discriminating specific category from the others. The ROC curve and the corresponding area under ROC curve (AUC) for each category were calculated in each model using the python library sklearn. Differences between various AUCs were compared using a Delong test. 95% CIs for sensitivity and specificity were calculated with the Clopped-Pearson method. The diagnostic likelihood ratio (DLR) was calculated to evaluate the clinical value of binary models. All statistical tests were two-sided with a significance level of 0·05.

## 3 Results

### 3.1 Baseline characters

Between Jan, 2014, and Dec, 2018, 2942 patients with liver tumors were enrolled from the hepatic focal lesions MR imaging database at SRRSH (**Figure 1**). Owing to undetermined final diagnosis and prior anti-tumor treatment before MRI inspection, 1260 patients were excluded. After quality control evaluation, 6077 of 47069 images were discarded because of poor quality or multi-sequence images that were not registered during image processing. For the internal independent validation dataset, 5172 tumor images from 220 patients were included at SRRSH between Jan, 2019, and Jul, 2019. At the two other participating hospitals, 2916 images of 173 patients were obtained from Hangzhou First People’s Hospital and 1338 images of 72 patients were acquired from Zhejiang Tongde Hospital. The patient characteristics were summarized in **Table 1**. Detailed diagnosis information about each type of tumors in training and validation sets was shown in **Supplementary Table S2**.

**Table 1:** Baseline characteristics.

Characteristic	Training Set	Validation Set (n=465)			p value
	SRRSH (n=1494)	SRRSH (n=220)	HZFPH (n=173)	TDH (n=72)	
**Age**	52 (13.41)	55 (14.98)	54 (13.49)	55 (15.09)	0.003
**Gender** ** Male** ** Female**	811 (54.3%) 683 (45.7%)	132 (60.0%) 88 (40.0%)	92 (53.2%) 81 (46.8%)	42 (58.3%) 30 (41.7%)	0.378
**Abscess**	73 (4.89%)	12 (5.45%)	12 (7.10%)	6 (8.33%)	0.400
**Cyst**	155 (10.37%)	17 (7.73%)	22 (13.02%)	1 (1.39%)	0.027
**Hemangioma**	275 (18.41%)	29 (13.18%)	29 (17.16%)	23 (31.84%)	0.005
**FNH**	197 (13.19%)	29 (13.18%)	14 (8.28%)	6 (8.33%)	0.205
**Benign nodules**	150 (10.14%)	29 (13.18%)	14 (8.28%)	4 (5.56%)	0.206
**Metastatic malignancy**	140 (9.37%)	36 (16.36%)	22 (13.02%)	13 (18.36%)	0.002
**HCC**	396 (26.51%)	56 (24.45%)	42 (24.85%)	16 (22.22%)	0.832
**Other primary malignancy**	108 (7.23%)	12 (5.45%)	14 (8.28%)	3 (4.17%)	0.524
**Lesion diameter (mm)**	49.48 (35.39)	48.03 (34.78)	41.49 (24.87)	40.06 (29.04)	0.016

Data are mean (SD) or n (%). SRRSH, Sir Run Run Shaw Hospital; HZFPH, Hangzhou First People’s Hospital; TDH, Zhejiang province Tongde Hospital. p<0·05 indicates that patient age and sex composition or the proportion of each category varied significantly by hospital (the Kruskal-Wallis H test was used to test whether patient age varied significantly by hospital, and the χ² test was used to test whether sex composition or the proportion of each category varied significantly by hospital).

### 3.2 AUC performance of CNN models

**Figure 2:**
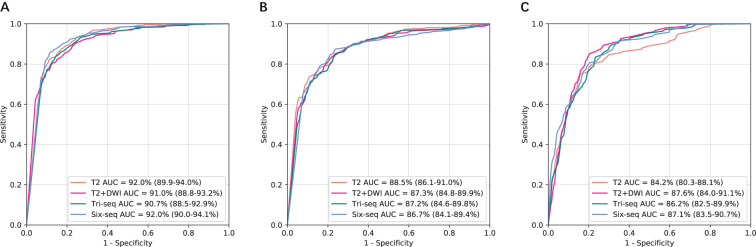
Comparison of receiver operating characteristic curves between T2, T2+DWI and Tri-Seq, Six-Seq models for the assessment of three categories in the independent SRRSH internal validation cohort. **(A)** Benign tumor versus malignancy. **(B)** Primary malignancy versus other lesions(benign and metastatic tumors). **(C)** Metastatic malignancy versus other lesions (benign and primary malignant tumors). SRRSH, Sir Run Run Shaw Hospital; AUC, area under the receiver operating characteristic curve; Tri-Seq, Three sequences; T2+DWI+Pre-contrast T1; Six-Seq, Six sequences; T2+DWI +Pre-contrast T1+ late arterial, portal venous, equilibrium phase.

The CNN models were first validated on the internal independent SRRSH dataset (**Figure 2**). T2 and T2+DWI exhibited similar performance compared to the other two multi-sequence models in classifying benign tumor, primary malignancy and metastatic tumor. Compared with T2+DWI, the other three models for each category bascially showed no statistical significance in AUC (p>0.05, **Supplementary Table S3**). However, the ability for distinguishing metastatic tumor was significantly inferior in T2 model compared to T2+DWI model (p=0.03, **Supplementary Table S3**). The AUCs of T2+DWI reached 0.91, 0.873, and 0.876 for three categories, respectively, while in T2 model, the AUCs were 0.92, 0.885 and 0.842.

**Figure 3:**
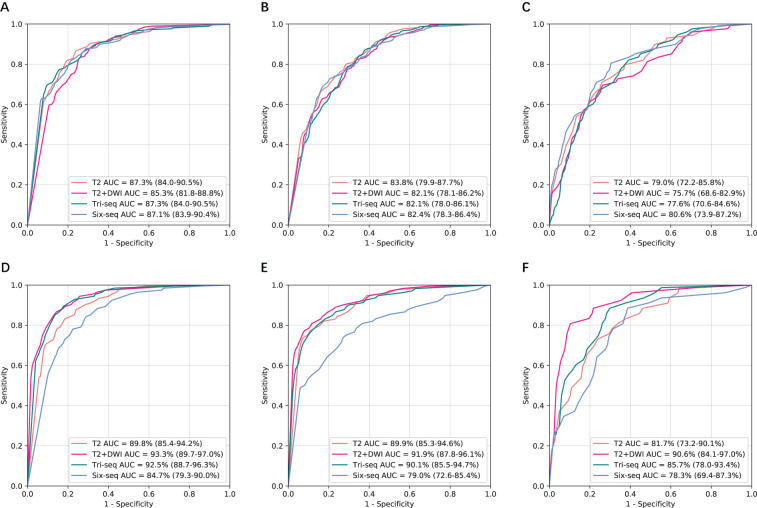
Comparison of receiver operating characteristic curves between T2, T2+DWI and Tri-Seq, Six-Seq models for the classification of three categories in two external validation cohorts.**(A, D)** Benign tumor versus malignancy. **(B, E)** Primary malignancy versus other lesions(benign and metastatic tumors). **(C, F)** Metastatic malignancy versus other lesions (benign and primary malignant tumors). **(A-C)** Hangzhou First People’s Hospital external independent validation set. **(D-F)** Zhejiang province Tongde Hospital external independent validation set. AUC, area under the receiver operating characteristic curve; Tri-Seq, Three sequences; T2+DWI+Pre-contrast T1; Six-Seq, Six sequences: T2+DWI +Pre-contrast T1+ late arterial, portal venous, equilibrium phase.

To further examine generalizability, we tested the models on the two external independent cohorts beyond the SRRSH data (**Figure 3**). The AUCs on these two test datasets presented with similar trends to SRRSH validation set. On the HZFPH dataset, the performances of four models on three-way classification were not statistically different (p>0.05, **Supplementary Table S3**). However, on the TDH dataset, the AUCs of T2+DWI were significantly better than Six-Seq model with enhanced images (p<0.01, **Supplementary Table S3**), which might be related to the different contrast medium used in TDH validation set and SRRSH training set. The corresponding ROC curves were shown in **Figures 3A–C** and **2D–F**.

### 3.3 Diagnostic accuracy of non-contrast models

**Figure 4:**
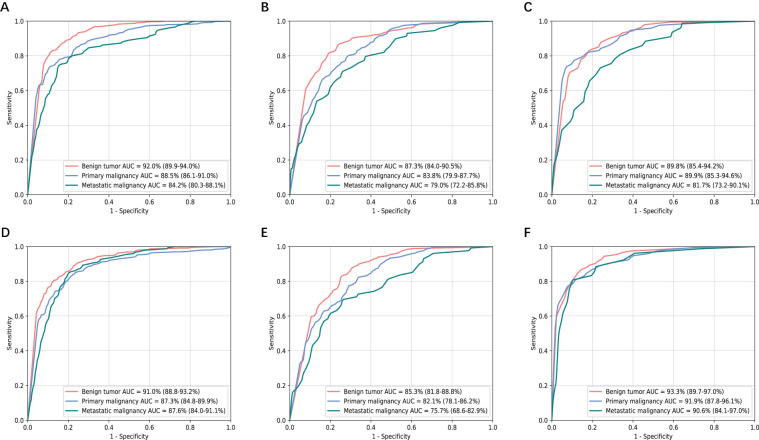
Receiver operating characteristic curves and area under the curve (AUC) analysis of two non-contrast models in three independent validation sets. **(A-C)** T2 model. **(D-F**) T2+DWI model. **(A, D)** Sir Run Run Shaw Hospital internal validation set. **(B, E)** Hangzhou First People’s Hospital external validation set. **(C, F)** Zhejiang province Tongde Hospital external validation set.

The performance of two models based on non-contrast images in classifying liver tumors on three independent validation datasets was shown in **Figure 4**. Their diagnostic accuracy showed no significant variation for classifying benign tumor and primary malignant tumor (P>0.05, **Supplementary Table S3**). However, T2+DWI exhibited a higher diagnostic accuracy compared with T2 for differentiating metastatic tumors from the other tumors, and differences of AUCs were all statistically significant (p<0.05, **Supplementary Table S3**) in SRRSH and TH datasets. The sensitivity and specificity analyses also demonstrated that T2+DWI was better than T2 (**Supplementary Table S4**) based on the comprehensive consideration about their performance on the three hospital datasets.

We examined the internal features learned by the CNNs of non-contrast models using t-SNE (t-distributed Stochastic Neighbor Embedding) (32) (**Supplementary Figure S2**). Each point represented a tumor image projected from the high-dimensional output of the CNN’s last hidden layer into two dimensions. The point cluster of benign tumors were basically split from those of malignant tumors, while the point clusters of two malignant categories were partly mixed. This indicated that the CNN could distinguish malignant images from benign images with a high accuracy, while more prediction errors occurred within the specific classifications of malignant tumors.

**Figure 5:**
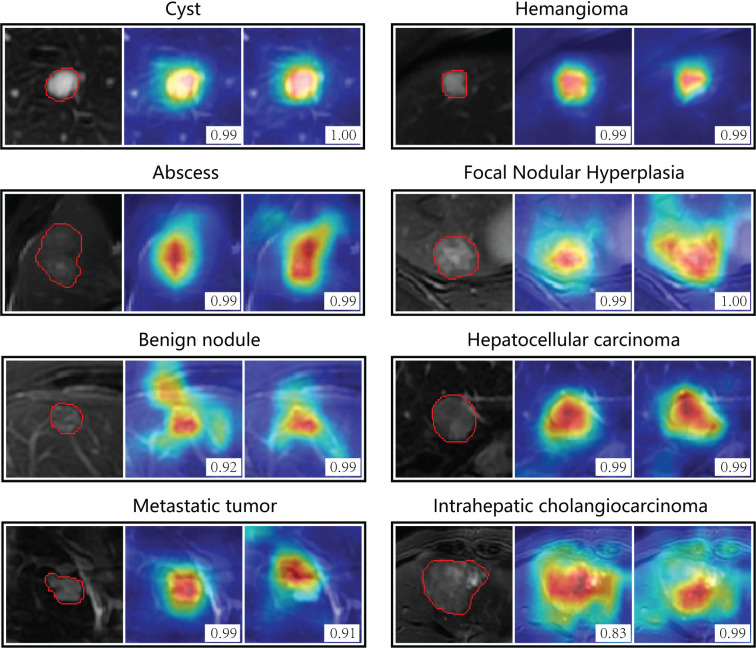
Attention maps of non-contrast models on eight types of focal liver lesions. The color-coded maps highlight regions which were most discriminative for a certain category. Red indicates the areas that contributed most, and blue areas contributed least. The left column is the region of interest from T2 image, the middle is the attention map of T2 model, and the right is attention map of T2+DWI model. The number in the picture indicates the probability of corresponding category predicted by the model. The original T2 images for each lesion were presented in **Supplementary Figure S4**.

**Figure 5** showed attention maps from eight types of cases to interpret the diagnostic mechanism of the neural networks. These lesions were difficult to distinguish on T2 by naked vision, while CNN models provided accurate diagnostic outcomes. The map quantified each pixel’s contribution to diagnosis by analyzing the lesion ROI. The red parts indicated areas that provided more related information during the network’s diagnostic process. The networks focused most of its attention on the tumor lesions themselves and ignored liver background.

### 3.4 Binary classification at the specific algorithm

In the performance analysis above, the prediction results about CNN models were all based on single 2-D MR image slice of liver tumor lesions. However, in clinical setting, one lesion with several image slices usually had only one diagnosis. Therefore, we tried to develop an algorithm which could combine the confidences at slice-level to predict the lesion-level confidence.

Firstly, the predicted vectors of all slices for each lesion were summed up and the category with the largest value was taken as the final diagnosis of the lesion. Then we obtained the diagnostic performance of the T2+DWI model on three independent test sets. Our study also conducted performance comparisons with radiologists. To ensure a fair comparison, the radiologists only relied on the T2 and DWI sequences to make independent diagnoses, while blinded to medical history and histopathological/radiological reports.

**Figure 6:**
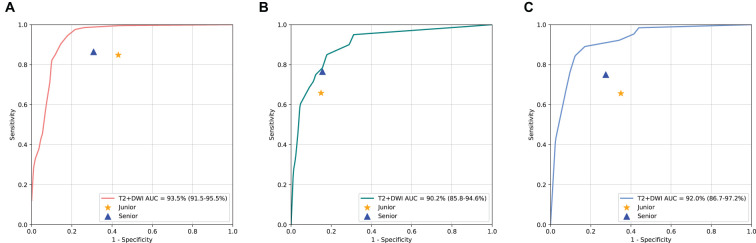
Performance Comparison of T2+DWI model with Radiologists on three dependent test sets. ROC curves for binary classification; **(A)** Sir Run Run Shaw Hospital internal validation set. **(B)** Hangzhou First People’s Hospital external validation set. **(C)** Zhejiang province Tongde Hospital external validation set.

The ROC curves depicted in **Figure 6** highlighted that T2+DWI model surpassed all radiologists in binary classification on three independent test sets, achieving an AUC of 0.935 (95% CI: 0.915-0.955), 0.902(0.858-0.946), 0.920(0.867-0.972). In particular, the model achieved superior performance in terms of accuracy, sensitivity, and specificity. The accuracy across three test sets was significantly higher than that of junior radiologists (P<0.05) and was comparable to that of senior radiologists. The sensitivities from the model were 0.908, 0.882, and 0.843, respectively. While superior to those of radiologists, these differences did not reach statistical significance.

In order to reduce the risk of delayed or missed care from false negatives, we further defined an algorithm as follows: the tumor was classified as benign only if all the related 2D slices were predicted negative, otherwise, once any of the slices was predicted as primary or metastatic malignancy, the tumor should be classified as malignant. **Table 2** presented the sensitivity and specificity of T2 and T2+DWI model based on this rule, along with the corresponding positive and negative DLRs.

**Table 2:** Sensitivity, specificity, and diagnostic Likelihood Ratio testing associated with T2 and T2+DWI models at specific algorithm.

Variable	SRRSH							
	T2				T2+DWI			
	Sensitivity, %	Specificity, %	DLR+	DLR-	Sensitivity, %	Specificity, %	DLR+	DLR-
**Malignancy**	95.2 (89.0-98.4)	68.2 (58.6-76.7)	2.99 (2.15-4.23)	0.07(0.02-0.19)	98.1 (93.2-99.8)	70.0 (60.5-78.4)	3.27 (2.36-4.61)	0.03 (0.00-0.11)

	HZFPH							
	T2				T2+DWI			
	Sensitivity, %	Specificity, %	DLR+	DLR-	Sensitivity, %	Specificity, %	DLR+	DLR-
**Malignancy**	81.8 (71.4-89.7)	70.0 (59.4-79.2)	2.73 (1.76-4.31)	0.26 (0.13-0.48)	89.7 (85.9-92.7)	73.3 (63.0-82.1)	3.21 (2.05-5.18)	0.19 (0.09-0.38)

	TDH							
	T2				T2+DWI			
	Sensitivity, %	Specificity, %	DLR+	DLR-	Sensitivity, %	Specificity, %	DLR+	DLR-
**Malignancy**	87.5 (71.0-96.5)	72.5(56.1-85.4)	3.18 (1.62-6.61)	0.17 (0.04-0.52)	87.5 (71.0-96.5)	75.0 (58.8-87.3)	3.5 (1.72-7.60)	0.17 (0.04-0.49)

DLR, diagnostic likelihood ratio; SRRSH, Sir Run Run Shaw Hospital; HZFPH, Hangzhou First People’s Hospital; TDH, Zhejiang province Tongde Hospital. The sensitivity, specificity, and DLRs for three independent validation datasets at the specific algorithm are shown. The algorithm is defined that when all the images of one patient are benign judged by the CNN model, the case is classified to benign tumor, in contrast, this case is classified to malignancy even if only one image indicated malignant. This algorithm can provide the greatest sensitivity for suspicious malignancy while maintaining an adequately high specificity for benign tumors as to reduce the use of contrast agents.

The sensitivities of malignant tumors gained from T2 model were 95.2%, 81.8%, and 87.5% in SRRSH, HZFPH and TDH datasets, respectively, and the corresponding results in T2+DWI model were 98.1%, 89.7%, and 87.5% respectively, while all specificities were almost greater than 70%. These results indicated that our models could identify over 95% patients with malignancy at best using non-contrast images, and more than 70% of patients with benign tumors could have the opportunity to avoid a further inspection using contrast mediums.

## 4 Discussion

In this multicenter study, we investigated whether different categories of liver tumor could be differentiated by deep learning CNN models using only non-contrast MRI. Compared with the multi-sequence model using enhanced images, Model T2 and T2+DWI showed similar performances on classifying liver masses into benign liver tumors, primary malignancy and metastatic malignancy. Their robustness and generality were demonstrated in three independent validation datasets. Moreover, under the defined algorithm, they could identify more than 98% malignancy and over 70% benign lesions at best. To the best of our knowledge, this is currently the largest study in the field of deep-learning-assisted liver tumor diagnosis based on non-contrast MR images worldwide, which has the most variable types of focal liver lesions.

To date, there are hardly few studies that explore the feasibility of non-contrast MRI for classifying liver tumors using deep learning (27, 33). Previous studies are usually based on enhanced images and smaller datasets (n< 500 patients). In contrast, our model exhibited a robust performance on multiple independent, real-world, heterogeneous datasets (acquired with many different imaging protocols and scanners, **Supplementary Table S1**), independent of differences in patient demographics. Although the six-seq model added with enhanced images did not perform better than the non-contrast models on validation sets, it should still be noted that its performance might be underestimated owing to the incomplete registration between enhanced sequences and non-enhanced sequences. Especially in the TDH set, the performance of the six-sequence model was significantly weaker than the T2 model. It might result from different contrast media (**Supplementary Table S1**) used in Tongde hospital (Gadodiamide, 0.1mmol/kg) and SRRSH (Gadopentetate dimeg-lumine, 0.2 mmol/kg) that lead to different enhanced-image features in TDH validation set compared with the training set.

As for non-contrast models in this study, T2+DWI showed similar diagnostic efficacy with T2 for classifying liver tumors. However, given that the excellent performance of DWI sequence in detecting small malignant lesion (<2cm) (34), T2+DWI should be the better choice in clinical application. After all, the problem of automatic tumor detection was not considered in this study. Moreover, for the TDH validation set with high-quality DWI images, the performance of T2+DWI was better than that of T2 model, in contrast, the results of the other two larger datasets with worse diffusion images were not improved. These results indicated that the performance of T2+DWI model was highly associated with the quality of diffusion images.

For three-way classification per image, it is commonly seen that differentiating metastatic malignancy from the other two categories is more challenging in non-contrast models. Many metastatic tumors were mis-predicted primary malignancy. This is because the heterogeneity of metastatic lesions is more severe owing to diverse primary tumor sites and histological types, and the proportion of this category (11.5%, 4836/42150) was much less than that of images with other categories in the training set. The current CNN models still have tremendous room for improvements, and it is likely that CNN may achieve better sensitivity in assessing metastatic tumors, if the sample population of this category could be further extended in future studies. Similarly, this situation has also been observed in some rare types of two other categories, such as adenoma, ICC, small highly differentiated HCC, etc., which were more likely to be misclassified.

The non-contrast models achieved basically satisfactory diagnosis accuracy at image-level, encouraging further exploration of its utility at patient-level. According to our defined rule, over 95% of patients with malignant tumors in SRRSH validation set were correctly judged, with a bit inferior about this indicator in two other datasets. However, an inspection of misclassifications also provided excellent feedback for our models (see **Supplementary Figure S3**, confusion matrix). These errors are mostly concentrated in the intrahepatic cholangiocarcinoma without typical image manifestations, and usually all images of the lesion are misjudged. For example, two ICCs from HFPH were considered as inflammatory granuloma and epithelial hemangioendothelioma respectively in formal radiology reports. These tumors were underrepresented in the training set and typically had a benign-looking appearance.

To the best of our knowledge, this is the largest multicenter study that aimed to analyze the diagnostic performance of non-contrast MRI for liver tumors by means of deep neural networks, covering the most variable types of focal liver lesions. This system could be applicable to get the first-step judgement for patients with liver masses by non-contrast MRI, and then potential malignant patients be selected for further enhanced inspections with suitable contrast agents. It could be beneficial especially for the patients that require multiple follow-up MRIs, such as those with benign lesions, or at relatively high risk of liver metastasis, or post liver cancer resection, etc., which can avoid unnecessary enhanced testing to reduce side effects and financial costs.

The work presented here has limitations. First, as our study population was composed of those have confirmed focal liver lesions (usually >1cm), our study results should be interpreted with caution. Future studies need to involve more patients with <1cm small lesions, especially those at high risk of HCC or metastasis. Prospective studies focusing on these specific populations will be more convincing. Second, our study performed in a diagnostic setting, thus the detection ability to lesions under non-contrast MRI needs to be further demonstrated. Fortunately, some studies have provided optimistic evidence. Non-contrast MRI showed high sensitivity and specificity for detecting HCCs in the early stage and in high-risk HCC patients under the evaluation of radiologists (11, 12, 14, 35, 36). In the study of Kim et al (26), a fully automated deep learning model outperformed less experienced radiologists in detecting very small HCCs using hepatobiliary phase MR images. From this perspective, we have reason to believe that the deep learning model using T2 and DWI images may also have a higher detection performance than human readers. Third, future studies need to involve more patients of small number of types in a large scale, as well as to achieve an equal distribution of patients in major categories, to make the deep learning model better trained. Moreover, the model itself also needs to be further developed with more comprehensive integration of other clinical data, such as medical history, tumor markers, other serological results, etc., which are valuable for tumor diagnosis.

In summary, using DL algorithms, NC-MRI provided accurate diagnosis for liver tumors in classifying to benign, primary malignancy and metastatic tumors. Moreover, the sensitivity of malignant tumors achieved significant improvement at the patient-level algorithm. In the independent internal and external cohorts, the models also showed excellent robustness. The developed DL model has potential to be used for benign tumors follow-up, surveillance of HCC and liver metastasis that need regular repetitive examinations in high-risk patients, yet further prospective studies are still needed before applied to real-world clinical settings.

## Data Availability

The data analyzed in this study is subject to the following licenses/restrictions: The original contributions presented in the study are included in the article/**Supplementary Material**. Further inquiries can be directed to the corresponding authors. Requests to access these datasets should be directed to 11718287@zju.edu.cn.
