# Supplementary material for: Deep learning-assisted diagnosis of liver tumors using non-contrast magnetic resonance imaging: a multicenter study
Source: Front Oncol. 2025 Jul 10;15:1582322. doi: 10.3389/fonc.2025.1582322 (PMC12287020; doi:10.3389/fonc.2025.1582322)
Supplement: Supplementary file 1 [file DataSheet1.pdf]

Supplementary Material

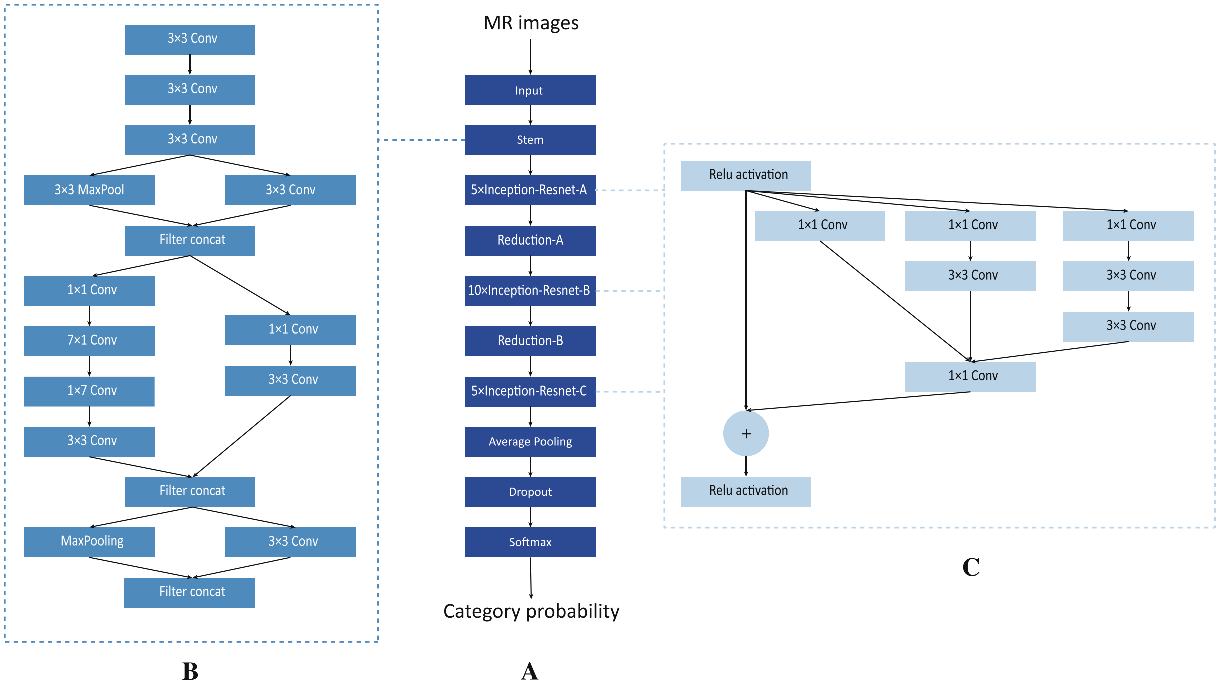

Figure S1. Schematic of the deep learning algorithm. The input of the model is MRI image of the liver tumor, while the output is the predicted probability of each category. (A) The major architecture of convolutional neural network. (B) Details of Stem module. (C) Details of Inception-Resnet A/B/C module.

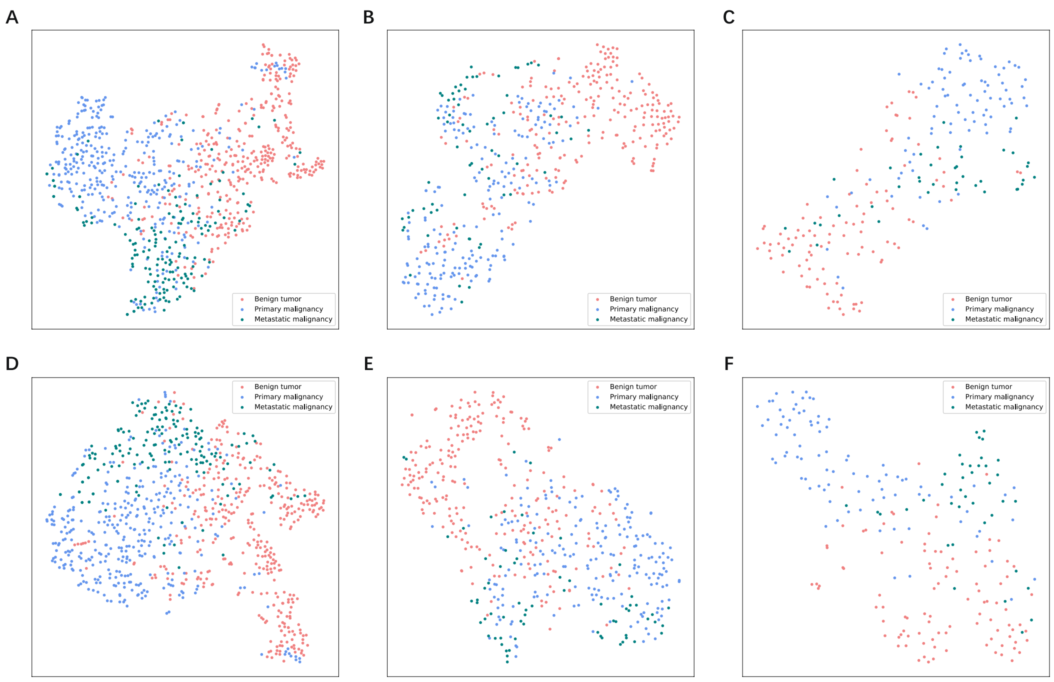

Figure S2. Illustration of non-contrast models learned by deep-learning, which projected to 2 dimensions for visualization via the t-SNE algorithm using values of the last fully connected layer in the CNNs of three validation sets. Scatterplots where each point represents an image of lesions and the color represents the true category, show how the algorithm clusters. (A-C) T2 model. (D-F) T2+DWI model. (A, D) Sir Run Run Shaw Hospital internal validation set. (B, E) Hangzhou First People's Hospital external validation set. (C, F) Zhejiang province Tongde Hospital external validation set.

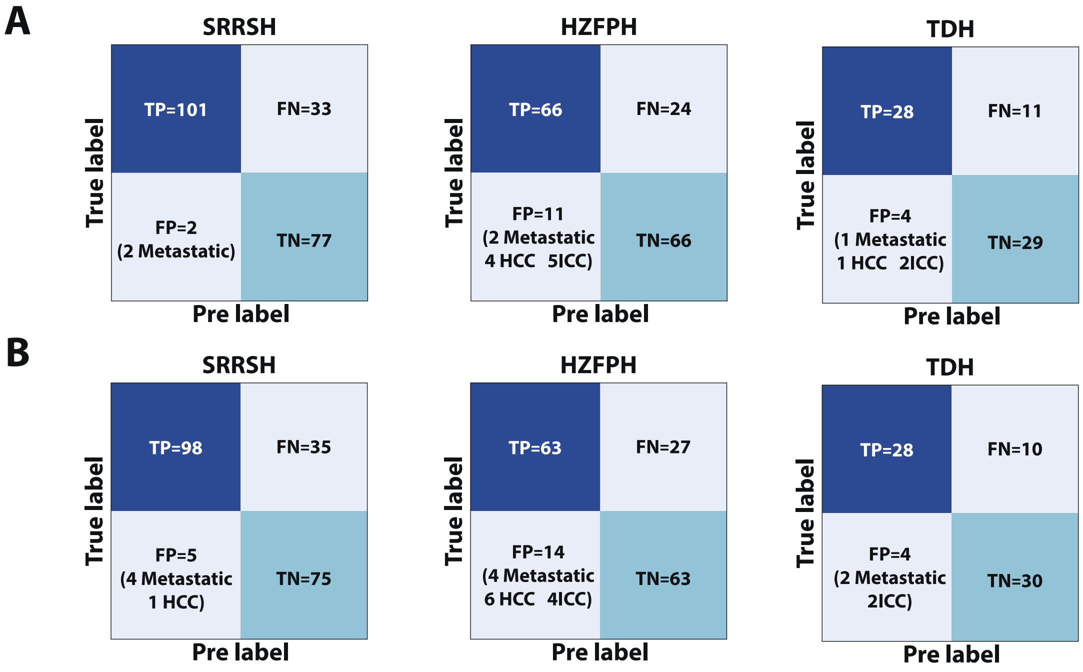

Figure S3. Confusion tables showing the binary-classification results of non-contrast models at patient-level among three validation sets. TP=true positive; FP=false positive; FN=false negative; TN=true negative.

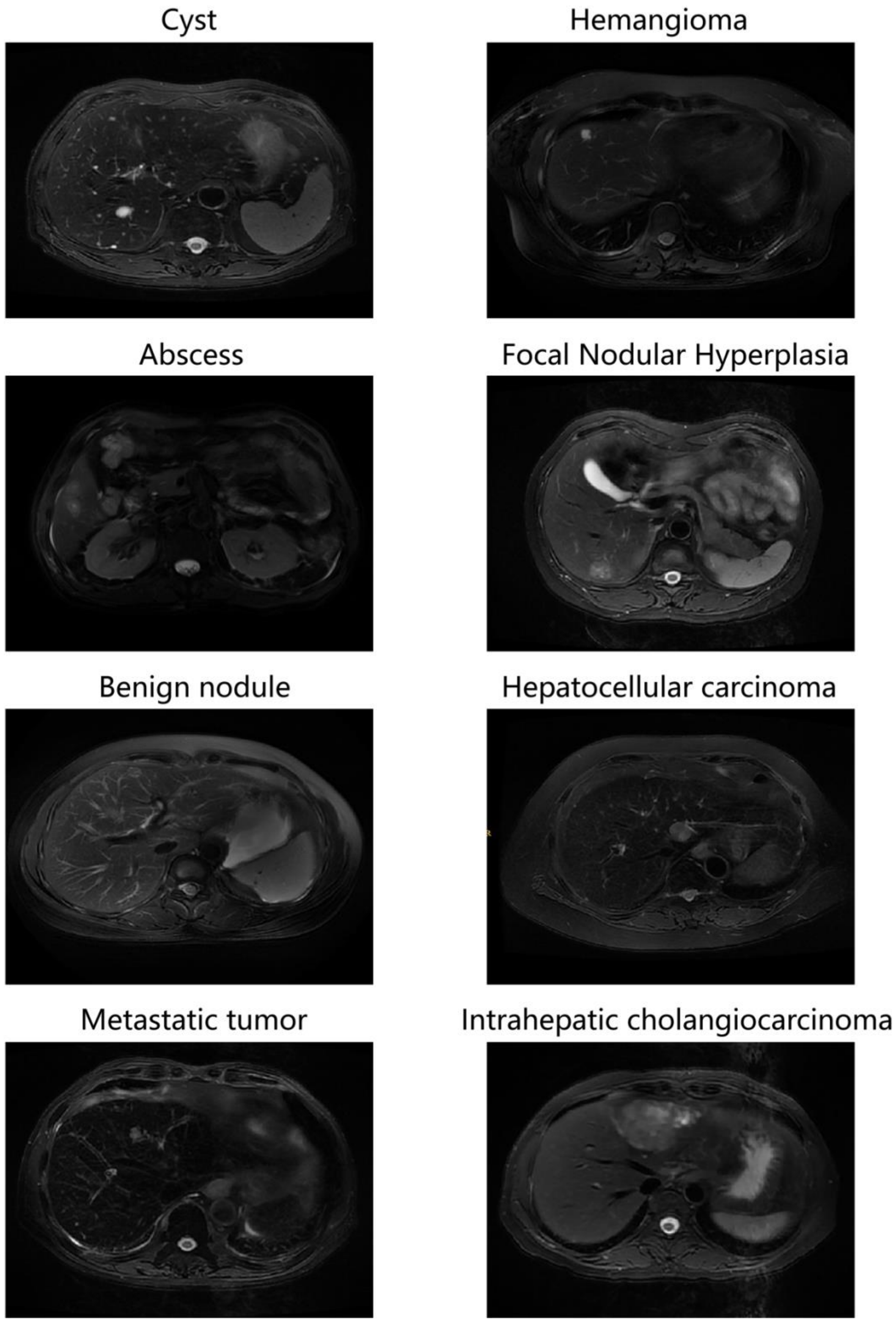

Figure S4. The original T2 images of eight types of liver tumors.

Table S1. The MR scanners and contrast mediums used in three hospitals

	SRRSH	HZFPH	TDH
MR scanner	GE MR Singna HDX 3.0T<br><br>GE MR Singna HD Excite scanners 1.5T<br><br>Simen MR Skyra 3.0T<br><br>Simen magnetom avan-to Dot1.5T<br><br>GE discovery MR 750 scanners.	Siemens AG<br><br>Magnetom Verio 3.0T<br><br>GE Singna Explore 1.5T	Siemens AG<br><br>Magnetom Verio 3.0T
Contrast medium	Gadopentetate dimeg-lumine (Bayer)<br><br>0.2 mmol/kg<br><br>(most patients）<br><br>Gadoxetic acid disodium 0.1 mmol/kg （very few patients）	Gadopentetate dimeglumine (CONSUN,Guangzhou)<br><br>0.2 mmol/kg	Gadodiamide（GE ）<br><br>0.1 mmol/kg<br><br>（most patients）<br><br>Gadopentetate dimeglumine<br><br>(Beilu, Beijing)<br><br>0.2 mmol/kg

SRRSH=Sir Run Run Shaw Hospital. HZFPH=Hangzhou First People’s Hospital. TDH=Zhejiang province Tongde Hospital.

Table S2. Diagnostic details for each type of liver tumors

Parameter	**Training Set** (number of patients)		**Validation Set**					
			SRRSH		HZFPH		TDH	
	YES	NO	YES	NO	YES	NO	YES	NO
**Abscess**	27 (58.7%)	46 (41.3%)	2 (16.7%)	10 (83.3%)	0 (0.0%)	12 (100%)	0 (0.0%)	6 (100%)
**Cyst**	18 (11.6%)	137 (88.4%)	2 (11.8%)	15 (88.2%)	0 (0.0%)	22 (100%)	0 (0.0%)	1 (100%)
**Hemangioma**	77 (28.0%)	198 (72.0%)	3 (10.3%)	26 (89.7%)	0 (0.0%)	29 (100%)	0 (0.0%)	23 (100%)
**FNH**	46 (23.3%)	151 (76.7%)	5 (17.2%)	24 (82.8%)	0 (0.0%)	14 (100%)	0 (0.0%)	6 (100%)
**Other BN**	55 (36.7%)	95 (63.3%)	5 (17.2%)	24 (82.8%)	1 (7.1%)	13 (92.9%)	0 (0.0%)	4 (100%)
**HCC**	384 (96.7%)	12 (3.3%)	39 (69.6%)	17 (30.4%)	40 (95.2%)	2 (4.8%)	7 (43.7%)	9 (56.3%)
**Metastasis**	140 (100%)	0 (0.0%)	16 (44.4%)	20 (55.6%)	14 (63.6%)	8 (6.4%)	4 (30.8%)	9 (69.2%)
**Other primary malignancy**	108 (100%)	0 (0.0%)	12 (100%)	0 (0.0%)	14 (100%)	0 (0.0%)	2 (66.7%)	1 (33.3%)

Data are n (%). YES=Patients diagnosed by histopathologic reports; NO= Patients diagnosed based on a combination of imaging and clinical data; FNH= focal nodular hyperplasia; Other BN= Other benign nodules; HCC= Hepatocellular carcinoma; SRRSH=Sir Run Run Shaw Hospital. HZFPH=Hangzhou First People’s Hospital. TDH=Zhejiang province Tongde Hospital.

Table S3. Delong Test about receiver operating characteristic curves between T2+DWI and other models about each category in three validation sets

Model	P Value								
	SRRSH			HZFPH			TDH		
	Benign	Primary	Metastatic	Benign	Primary	Metastatic	Benign	Primary	Metastatic
T2	0.287	0.192	0.031	0.178	0.222	0.310	0.069	0.210	0.023
Tri-Seq	0.679	0.859	0.280	0.179	0.988	0.475	0.647	0.276	0.039
Six-Seq	0.289	0.596	0.782	0.283	0.884	0.209	0.001	$1.72*10^{-5}$	0.008

Tri-Seq=Three sequences: T2+DWI+Pre-contrast T1; Six-Seq=Six sequences: T2+DWI +Pre-contrast T1+ late arterial, portal venous, equilibrium phase;

SRRSH=Sir Run Run Shaw Hospital. HZFPH=Hangzhou First People's Hospital. TDH=Zhejiang province Tongde Hospital.

Table S4. Diagnostic performance of T2 and T2+DWI for three-way classification in three independent validation sets.

**Validation datasets**	**Models**	**Benign tumors**		**Primary malignancy**		**Metastatic malignancy**	
		**Sensitivity,%**	**Specificity,%**	**Sensitivity,%**	**Specificity,%**	**Sensitivity,%**	**Specificity,%**
**SRRSH**	T2	85.3 (81.1, 88.8)	84.3 (80.8, 87.3)	79.2 (74.5, 83.4)	81.0 (77.4, 84.3)	41.7 (34.1, 49.7)	94.1 (92.1, 95.8)
	T2+DWI	83.0 (78.7, 86.8)	83.3 (79.8, 86.4)	81.5 (77.0, 85.5)	79.7 (75.9, 83.0)	36.8 (29.4, 44.7)	94.7 (92.8, 96.3)
**HZFPH**	T2	84.9 (79.7, 89.3)	76.8 (71.1, 81.8)	68.4 (61.3, 75.0)	81.8 (76.9, 86.0)	31.3 (20.2, 44.1)	93.8 (91.1, 95.9)
	T2+DWI	82.3 (76.8, 87.0)	75.2 (69.4, 80.3)	63.2 (55.9, 70.0)	83.1 (78.3, 87.1)	34.4 (23.0, 47.3)	90.5 (87.3, 93.1)
**TDH**	T2	80.6 (71.4, 87.9)	83.2 (75.5, 89.3)	82.6 (72.9, 89.9)	77.4 (69.5, 84.1)	33.3 (19.1, 50.2)	95.7 (91.6, 98.1)
	T2+DWI	85.7 (77.2, 92.0)	87.2 (80.1, 92.5)	82.6 (72.9, 89.9)	85.4 (78.4, 90.9)*	59.0 (42.1, 74.4)*	95.1 (90.9, 97.7)

Statistical quantifications were demonstrated with 95% CI; *P<0.05, inter-model comparison using the McNemar's test. SRRSH=Sir Run Run Shaw Hospital. HZFPH=Hangzhou First People's Hospital. TDH=Zhejiang province Tongde Hospital.

Table S5. Diagnostic performance of T2+DWI and radiologists for patient-wise binary classification in three independent validation sets.

Validation datasets	Malignancy	T2+DWI model	Senior Radiologist	Junior Radiologist	T2+DWI vs Sensior (P value)	T2+DWI vs Junior (P value)
**SRRSH**	**Accuracy,%**	0.901(0.838-0.941)	0.778(0.700-0.841)	0.709(0.627-0.781)	0.063	0.005
	**Sensitivity,%**	0.909 (0.839-0.978)	0.864 (0.781-0.946)	0.848(0.762-0.935)	0.547	0.008
	**Specificity,%**	0.892(0.825-0.897)	0.692(0.527-0.734)	0.569(0.448-0.689)	0.479	0.715
**HZFPH**	**Accuracy,%**	0.890(0.833-0.929)	0.817(0.8751-0.869)	0.768(0.687-0.818)	0.100	0.007
	**Sensitivity,%**	0.882(0.778-0.979)	0.765(0.801-0.948)	0.658(0.558-0.759)	0.265	0.425
	**Specificity,%**	0.898(0.776-0.926)	0.844(0.718-0.970)	0.851(0.789-0.939)	1.000	0.793
**TDH**	**Accuracy,%**	0.903(0.813-0.952)	0.736(0.624-0.824)	0.694(0.538-0.753)	0.056	0.039
	**Sensitivity,%**	0.843(0.780-0.873)	0.750(0.600-0.900)	0.656(0.600-0.800)	0.333	0.376
	**Specificity,%**	0.954(0.931-0.983)	0.725(0.567-0.863)	0.650(0.502-0.798)	0. 002	0.014

Statistical quantifications were demonstrated with 95% CI; *P<0.05, inter-model comparison using the McNemar's test. SRRSH=Sir Run Run Shaw Hospital. HZFPH=Hangzhou First People's Hospital. TDH=Zhejiang province Tongde Hospital.
